# Characteristics of Patients Undergoing Renal Artery Denervation in the United States

**DOI:** 10.1016/j.jscai.2026.105445

**Published:** 2026-06-02

**Authors:** Connor G. Oltman, Issam D. Moussa

**Affiliations:** a Carle Illinois College of Medicine, University of Illinois Urbana-Champaign, Urbana, Illinois; b Heart and Vascular Institute, Carle Health, Urbana, Illinois

**Keywords:** antihypertensives, electronic health records, renal denervation, resistant hypertension

## Introduction

Renal artery denervation (RDN) has recently entered routine clinical use in the United States after pivotal randomized clinical trials demonstrated durable reductions in blood pressure among patients with resistant hypertension.^1^, ^2^, ^3^ Although these studies established the safety and efficacy of RDN under controlled conditions, trial populations were highly selected, excluding many patients encountered in routine clinical practice. Therefore, this study aimed to describe the clinical profile and early outcomes of patients undergoing RDN in real-world practice in the United States.

## Methods

This retrospective cohort study used data from Epic’s Cosmos, a nationwide deidentified electronic health records database. Carle Institutional Review Board approval was not required as this study used aggregate deidentified data and was not considered human subjects research. Residents of the United States aged ≥18 years who underwent RDN between November 1, 2023, and March 31, 2025, were identified using Current Procedural Terminology (CPT) codes 0338T and 0339T.

For cohort characterization, age, sex, race and ethnicity, smoking status, and body mass index were obtained from encounter data at the time of RDN. Hypertension severity and comorbidities were identified using *International Classification of Diseases, Tenth Revision* (ICD-10) diagnosis codes documented in any clinical encounter in the 12 months preceding RDN. Antihypertensive medication use was defined using longitudinal prescription records during the same 12-month interval; medications with active prescription status at any time during the period, including those with a recorded end date within the interval, were included.

To evaluate early outcomes after RDN, analyses were restricted to patients with at least one clinical encounter in both a preoperative interval (3-6 months before RDN) and a postoperative interval (3-6 months after RDN). These prespecified windows were selected to capture recent preprocedural status and early follow-up while accommodating variability in real-world encounter timing and minimizing the immediate periprocedural period. Within each interval, the proportion of patients with active prescriptions for each antihypertensive medication class was calculated, and relative changes in prescription frequency were reported. Blood pressure response was assessed using systolic blood pressure (SBP) values documented during clinical encounters within the same intervals. The proportion of patients meeting an SBP target of <140 mm Hg during each interval was calculated, and the change in this proportion was reported overall and across prespecified subgroups. Blood pressure measurements and active prescriptions were summarized at the interval level and were not necessarily derived from the same encounter.

## Results

**Figure 1:**
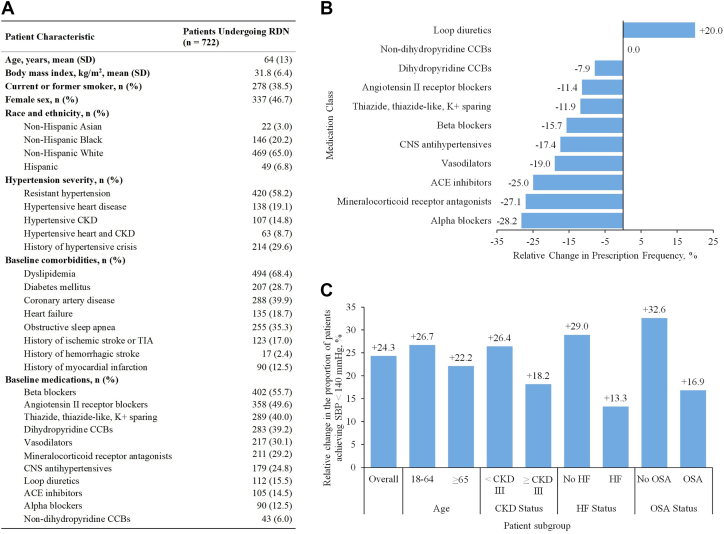
**Profile and outcomes of patients undergoing renal artery denervation in the United States.** (A) Demographic characteristics at the time of RDN, with hypertension severity, comorbidities, and antihypertensive medication use assessed during the 12 months preceding the procedure. (B) Relative change in prescription frequency for each medication class between the 3 to 6 month pre-RDN and 3 to 6 month post-RDN intervals. (C) Relative change in the proportion of patients achieving SBP <140 mm Hg between the 3 to 6 month pre-RDN and 3–6 month post-RDN intervals. ACE, angiotensin-converting enzyme; CCB, calcium channel blocker; CKD, chronic kidney disease; CNS, central nervous system; HF, heart failure; OSA, obstructive sleep apnea; RDN, renal artery denervation; TIA, transient ischemic attack.

This study identified 722 patients (mean age 64 ± 13 years; 46.7% female; 65.0% non-Hispanic White) who underwent RDN in routine clinical practice in the United States between November 1, 2023, and March 31, 2025 (Figure 1A). The mean body mass index was 31.8 ± 6.4 kg/m^2^, and 38.5% were current or former smokers. Overall, 58.2% of patients had documented resistant hypertension and 29.6% had a history of hypertensive crisis. Notable comorbidities included heart failure (18.7%) and obstructive sleep apnea (35.3%). In the year preceding RDN, the most prescribed medications were beta blockers (55.7%), angiotensin II receptor blockers (49.6%), and thiazide, thiazide-like, or potassium-sparing diuretics (40.0%).

In interval-based comparisons, the proportion of patients with active prescriptions decreased for most medication classes (Figure 1B). The proportion of patients meeting an SBP goal of <140 mm Hg was higher in the postprocedural interval (+24.3% overall), with similar findings observed across all subgroups (Figure 1C). The relative increase in the proportion of patients achieving SBP <140 mm Hg was lowest among those with heart failure (+13.3%), chronic kidney disease stage III or greater (+18.2%), and obstructive sleep apnea (+16.9%).

## Discussion

To our knowledge, this is one of the first studies to describe the real-world characteristics of patients undergoing RDN in US practice. Compared with trial populations, patients in this study were older, had a higher comorbidity burden, and were prescribed a wider range of antihypertensive agents. Limited insurance coverage during the early commercial adoption of RDN may have contributed to this age distribution, as Medicare-eligible patients may have had more consistent access than younger patients reliant on commercial insurance. The cohort exhibited a high mean body mass index and a high prevalence of obstructive sleep apnea, highlighting the importance of optimizing lifestyle and noninvasive management strategies before proceeding to procedural therapy. Additionally, a substantial proportion of patients had a documented history of hypertensive crisis, suggesting that RDN is being used in patients at particularly high risk for hypertension-related hospitalization.

After RDN, interval-based descriptive analyses demonstrated lower proportions of patients with active prescriptions for most antihypertensive medication classes and higher proportions meeting an SBP goal of <140 mm Hg during the postprocedural period. Smaller gains were observed among patients with obstructive sleep apnea, chronic kidney disease, or heart failure, conditions that may signal early nonresponse to RDN. Collectively, these findings suggest that RDN is being implemented among patients with complex, multifactorial hypertension and achieving favorable early outcomes in real-world practice.

This study has important limitations. The Cosmos database provides deidentified, aggregate-level data, precluding manual chart validation and patient-level adjudication; therefore, findings should be interpreted within the constraints of routinely collected electronic health record data. Additionally, Cosmos may not represent all patients undergoing RDN in the United States during the study period, as not all health systems use Epic, and participation in Cosmos is not universal among Epic-affiliated institutions. Accordingly, these findings should not be considered equivalent in scope or rigor to those derived from a dedicated national clinical registry. Comorbidities and hypertension severity were identified using ICD-10 diagnosis codes, which may be subject to misclassification or incomplete coding. The database structure did not permit identification of encounter-level timing or encounter frequency within each interval, including calculation of median post-RDN follow-up time. This is particularly relevant because the blood pressure effects of RDN may evolve over time. Interval comparisons of blood pressure may have been influenced by follow-up timing and surveillance bias if patients had more frequent follow-up after RDN than before the procedure. Finally, the comorbidities and medication use reported in Figure 1A were identified during the 12 months preceding RDN; therefore, treatment burden may be overestimated if medication regimens changed during that period. Clinical features were prespecified to mirror baseline characteristics from pivotal RDN trials and were limited to reliably captured structured data elements. In keeping with the descriptive intent of this study, analyses focused on cohort characterization and pre–post comparisons, and multivariable modeling was not performed.

## CRediT authorship contribution statement

**Connor G. Oltman:** Writing – review & editing, Writing – original draft, Visualization, Validation, Software, Methodology, Investigation, Formal analysis, Data curation, Conceptualization. **Issam D. Moussa:** Writing – review & editing, Supervision, Resources, Methodology, Investigation, Conceptualization.

## Declaration of competing interest

The authors declared no potential conflicts of interest with respect to the research, authorship, and/or publication of this article.
